# Antifungal Activity and Biocontrol Mechanism of *Fusicolla violacea* J-1 against Soft Rot in Kiwifruit Caused by *Alternaria alternata*

**DOI:** 10.3390/jof7110937

**Published:** 2021-11-04

**Authors:** Wenzhi Li, Youhua Long, Feixu Mo, Ran Shu, Xianhui Yin, Xiaomao Wu, Rongquan Zhang, Zhuzhu Zhang, Linan He, Tingting Chen, Jia Chen

**Affiliations:** 1 Research Center for Engineering Technology of Kiwifruit, Institute of Crop Protection, College of Agriculture, Guizhou University, Guiyang 550025, China; lwz9512@126.com (W.L.); gs.fxmo19@gzu.edu.cn (F.M.); s17708559157@126.com (R.S.); xhyin@gzu.edu.cn (X.Y.); xmwu@gzu.edu.cn (X.W.); zhuzhuzhang9612@126.com (Z.Z.); hln12396061028@126.com (L.H.); gzctt126@126.com (T.C.); c18184436145@126.com (J.C.); 2 Agricultural Industrial Park Management Committee in the East of Shuicheng County, Liupanshui 553000, China; zr73542@126.com

**Keywords:** antagonistic fungus, *Fusicolla violacea*, *Alternaria alternata*, kiwifruit, biocontrol

## Abstract

*Alternaria alternata* is the main pathogenic species of various crops, including kiwifruit (*Actinidia cinensis*). In this study, an antagonistic fungus, J-1, with high antifungal activity against *A. alternata* was isolated from *A. cinensis* “Hongyang.” The strain J-1 was identified as *Fusicolla violacea* via morphological identification and DNA sequencing. This study aimed to evaluate the antifungal activity and potential mechanism of the strain J-1 against *A. alternata*. The strain J-1 exhibited antifungal activity against *A. alternata*, with an inhibition rate of 66.1% in vitro. Aseptic filtrate (AF) produced by the strain J-1 could suppress the mycelial growth and conidia germination of *A. alternata* at the inhibition rates of 66.8% and 80%, respectively, as well as suppress the spread of *Alternaria* rot in fresh kiwifruit. We observed that many clusters of spherical protrusions appeared at the mycelial tips of *A. alternata* after treatment with 200 mL L^−1^ AF of J-1. Scanning electron microscopy analysis results showed that the mycelial structures were bent and/or malformed and the surfaces were rough and protuberant. Variations in temperature, pH, and storage time had little effect on the antifungal activity of the AF. Moreover, the AF could damage the integrity of cell membranes and cause intracellular content leakage. Meanwhile, the chitinase and β-1,3-glucanase enzyme activities increased significantly, indicating that the function of *A. alternata* cell wall was seriously injured. Eleven antimicrobial metabolites were identified by gas chromatography–mass spectrometry (GC–MS). The strain J-I and its AF exhibited well broad-spectrum antifungal activity against *Diaporthe eres*, *Epicoccum sorghinum*, *Fusarium graminearum*, *Phomopsis* sp., and *Botryosphaeria dothidea*, with inhibition rates ranging from 34.4% to 75.1% and 42.7% to 75.2%, respectively. *Fusicolla violacea* J-1 is a potential biocontrol agent against *A. alternata* and other fungal phytopathogens.

## 1. Introduction

Kiwifruit (*Actinidia* spp.), also known as Chinese gooseberry, is one of the most popular fruit worldwide owing to its high vitamin C content and abundant nutritional components [1]. However, with the rapid expansion of cultivation areas, kiwifruit production has been greatly affected by various diseases. Kiwifruit is prone to numerous pathogenic fungi. Studies have shown that *Phomopsis* sp., *Botryosphaeria dothidea*, *Botrytis cinerea*, *Alternaria alternata*, and *Pestalotiopsis microspora* are the main pathogens that cause kiwifruit postharvest rot, resulting in severe postharvest losses [2,3,4]. Among these pathogens, *Alternaria* is a widespread fungal genus that can cause severe economic losses to a variety of crops [5]. One of the most pathogenic species of this genus is *Alternaria alternata*, which causes fruit rot, leaf spot, or blight in important crops such as blueberries [6], cherries [7], tobacco [8], tomatoes [9], and sunflowers [10]. Furthermore, *A. alternata* can produce mycotoxins such as tenuazonic acid, alternariol, alternariol-monomethyl ether, and altenuene, which possess hematotoxic, genotoxic, and mutagenic activities [11]. Mycotoxins in food may be detrimental to human and animal health and cause safety concerns [12]. Puntscher et al. determined up to 9 of 17 *Alternaria* toxins in tomato sauce, sunflower seed oil, and wheat flour, which may impact on consumers’ health [13]. Yekeler et al. observed the esophageal dysplasias of rats after treatment with tenuazonic acid (TeA) produced by *Alternaria* species [14]. Studies have shown that the outbreak of onyalai, a human hematologic disorder disease in Africa, is related to tenuazonic acid [15]. A previous study also suggested that *A. alternaria* may be the cause of esophageal cancer in Lixian, China [16].

Our previous study showed that *Alternaria alternata* is the key fungal pathogen that causes the decay of *Actinidia cinensis* “Hongyang” and can enter the kiwifruit umbilical tissue in the early stages of growth and cause premature fruit drop. The pathogen quickly infects the inside of the fruit and causes fruit rot. Therefore, it is important to find effective and eco-friendly agents to manage this disease. To date, the main strategies for controlling plant diseases have relied on chemical control. Synthetic fungicides can significantly reduce the severity of disease; however, extensive and/or excessive use of fungicides usually leads to fungicide-resistant pathogen development, environmental pollution, and concerns regarding food safety. To deal with these issues, it is important to develop alternative antimicrobial agents that are both safe and eco-friendly.

Biological control is considered a safe and effective method to control the phytopathogens of various postharvest fruits [17]. In recent years, numerous reports have identified biological agents for controlling the postharvest diseases of fruit or vegetables. These include *Pseudomonas fluorescens* against soft rot disease in melon caused by *Erwinia carotovora* subsp. Carotovora [18], *Bacillus amyloliquefaciens* against *Pectobacterium carotovorum* in potatoes [19], *Lactobacillus plantarum* against *Pectobacterium carotovorum* subsp. *Carotovorum* on Chinese cabbage [20], and *Staphylococcus sciuri* against *Colletotrichum nymphaeae* in strawberries [21]. Besides their inhibitory activity on pathogen growth, many biological control agents exhibit their biocontrol capacity by inducing plants to acquire systemic resistance and compete with pathogens for ecological niche [22]. Although biocontrol technology has been applied to many fruits and vegetables, no studies have been conducted on the control of kiwifruit soft rot using *Fusicolla violacea*.

In the present study, we screened and identified an antagonistic fungus, *Fusicolla violacea* J-1, with strong antifungal activities from *Actinidia cinensis* “Hongyang.” The *Fusicolla violacea* (*Ascomycota*, *Hypocreales*, *Nectriaceae*) is synonymous with *Fusarium merismoides* var. *Violaceum* [23]. In general, most *Fusarium* sp. are pathogenic to plants; however, some endophytic *Fusarium* species has been reported as effective biocontrol agents against pathogens by inducing systemic resistance, competing for nutrients, or producing bioactive metabolites such as polyketides, alkaloids, terpenes, peptides, and steroids [24,25,26]. To date, there are no studies on the application of *Fusicolla violacea* for biological control. Based on this, the aseptic filtrate (AF) from strain J-1 was used to evaluate its inhibitory activity on the mycelial growth of *A. alternata*. Its biocontrol potential against *Alternaria* rot on kiwifruit was also evaluated. The changes in cell morphology of *A. alternata* were determined via scanning electron microscopy (SEM). The potential mechanism of antifungal activity was investigated by observing the effects of the AF on conidia germination, integrity of membranes, and activities of key enzymes in the cell walls of *A. alternata*. In addition, the broad-spectrum antifungal activity of the AF was tested against five pathogens. We also identified the metabolic compounds from *Fusicolla violacea* by gas chromatography–mass spectrometry (GC–MS). This study offers a new biological control resource against *A. alternata* and provides a theoretical basis for the better utilization of *Fusicolla violacea* to control other diseases in the future.

## 2. Materials and Methods

### 2.1. Pathogen and Antagonistic Strains

The pathogen *Alternaria alternata* (W-1) was provided by the Research Center for Engineering Technology of Kiwifruit, Guizhou University, China. The antagonistic strain *Fusicolla violacea* (J-1) was isolated from *Actinidia cinensis* “Hongyang,” which was collected from the orchard at Miluo Town in Shuicheng, Guizhou province, China (26°38′ N, 105°04′ E). The kiwifruit were first washed with sterile water to remove surface impurities and then sterilized as follows: sterile distilled water was applied to the fruit for 20 s, followed by 75% (*v*:*v*) ethanol application for 2 min. The fruit were then washed three times with sterile distilled water. After surface disinfection, the pericarp and pulp tissues were cut from the kiwifruit and transferred to potato dextrose agar (PDA) plates. After incubation at 28 °C for 7 d in the dark, colonies were inoculated to new PDA plates and incubated at 28 °C for 7 d. The pure colonies were soaked in 15% glycerol and stored at −80 °C until use.

### 2.2. Antagonistic Strain Screening

The isolated strains were tested in vitro for their antagonistic effect against *A. alternata* as per the method of Yan et al. [27] with small modifications. In brief, a 6-mm-diameter disk from the margin of each actively growing colony of *A. alternata* was transferred to the center of a PDA plate. Then, four similar-sized disks of the isolated strains were inoculated at four symmetrical points located 20 mm from the pathogen. Control tests were also performed using the pathogen alone. All plates were incubated at 28 °C for 5 d. The growth inhibition rate of mycelia was calculated by the following formula: i = (a_1_ − a_2_)/a_1_ × 100, where i is the growth inhibition rate of mycelia, a_1_ is the hyphae area of untreated pathogen, and a_2_ is the hyphae area of treated pathogen [28]. All experiments were performed in triplicate.

### 2.3. Antagonistic Strain Identification

The isolated antagonistic fungus was identified by morphology and DNA sequencing. The morphology of the mycelia and spores of fungi incubated at 28 °C for 10 d was observed under an optical microscope (LEICA ICC50 W, Leica Microsystems Co., Ltd., Shanghai, China). The morphological identification of the fungi was performed according to previous studies [29,30]. The genomic DNA of the antagonistic fungi was extracted using the BW-GD2416 Fungal gDNA Isolation Kit as per the manufacturer’s instructions. Primer sequences, 18S rDNA, and *β-tub* were selected with the relevant polymerase chain reaction (PCR) program to amplify the strain as per Watanabe et al. [31]. Amplification reactions with the primer pair for each gene were performed in a gcal cycler (T100^TM^ Thermal Cycler, Bio-Rad, Hercules, CA, USA). The amplified PCR products were sequenced by Sangon Biotech Co. Ltd. (Shanghai, China). The similarity of the resulting nucleotide sequences were analyzed using the NCBI BLAST tool. The multiple alignments of the sequences were performed at https://mafft.cbrc.jp/alignment/server/index.html (accessed on 15 April 2021). A polygene phylogenetic tree was constructed using the maximum likelihood (ML) method in MEGA 7.0 software with bootstrap values based on 1000 replications. *Neurospora crassa* OR74A was used as an outgroup.

### 2.4. Antifungal Activity of Aseptic Filtrate from Fusicolla violacea against Alternaria alternata

The effect of the AF on the mycelial growth of *A. alternata* was assessed according to a previous method [32] with minor modifications. In brief, three similar-sized disks of the antagonistic fungus were transferred to 0.25-L flasks containing 0.1 L potato dextrose broth (PDB). The flasks were placed on a constant temperature rotary shaker (150× *g* at 28 °C). After incubation for 7 d, the AF was collected by filtration through a sterile filter gauze, followed by filtration through a 0.22-μm membrane filter. The pathogen disks were then inoculated on the center of PDA plates containing 50 mL L^−1^, 100 mL L^−1^, and 200 mL L^−1^ (*v*:*v*) AF, respectively. After incubation for 7 d at 28 °C, the colony diameters were measured in two perpendicular directions. Sterile water was set as a negative control. All experiments were performed in triplicate. The inhibitory rate was calculated as follows: inhibitory rate (%) = [(d_control_ − d_treatment_)/d_control_] × 100, where d represents the diameter of the *A. alternata* colony.

### 2.5. Aseptic Filtrate Effects on the Conidial Germination of A. alternata

The conidial germination of *A. alternata* was assayed after treatment with the AF of the strain J-1. In brief, the AF was diluted to 100 mL L^−1^, 200 mL L^−1^, and 400 mL L^−1^ with sterile water and then mixed with the conidia suspension (1.0 × 10^7^ CFU mL^−1^) at a ratio of 1:1 (*v*:*v*) to make final AF concentrations of 50 mL L^−1^, 100 mL L^−1^, and 200 mL L^−1^, respectively. Then, 20 μL mixture was dripped onto the center of a concave slide and covered with a cover slip. The mixture was incubated in a Petri dish with moist filter paper at 28 °C for 12 h, and conidia germination was tested under an optical microscope. The germination was taken into account when the germ tube length exceeded half of the conidia’s length. Five fields were examined for each treatment to calculate the germination rate of conidia. The experiments were performed in triplicate, and sterile water was used as a negative control.

### 2.6. Antifungal Spectrum of the Strain J-1 and Its Aseptic Filtrate

To determine the broad-spectrum antifungal activity of the strain J-1, five pathogens were tested in this study. The pathogenic fungi *Diaporthe eres*, *Epicoccum sorghinum*, *Fusarium graminearum*, *Phomopsis* sp., and *Botryosphaeria dothidea* were provided by the Research Center for Engineering Technology of Kiwifruit, Guizhou University, Guiyang, China. The antagonistic activity of the strain J-1 was measured according to Section 2.2. The antifungal spectrum of the AF of the strain J-1 was tested by placing a 6-mm disk of the selected pathogens in the center of a PDA plate containing 200 mL L^−1^ AF. All experiments were performed in triplicate. After incubation for 7 d at 28 °C, the inhibitory rate was measured as described in Section 2.4.

### 2.7. Aseptic Filtrate Stability

The stability of the AF was assessed under the treatment of different thermal, acid–base, and storage time conditions as per a previous description [33] with minor modifications. The AF was prepared as described in Section 2.4. Aseptic filtrates were, respectively, treated at −80 °C, −20 °C, 0 °C, 20 °C, 80 °C, and 120 °C for 30 min and then cooled to room temperature for later use. The pH values were set from 4.0 to 11.0 for 30 min and then adjusted back to 7.0. The storage times were 0 d, 15 d, 30 d, and 45 d, respectively. After the different treatments, the effects of 200 mL L^−1^ AF on the mycelial growth inhibition of *A. alternata* were determined to evaluate the filtrate stability. All experiments were performed in triplicate.

### 2.8. Propidium Iodide (PI) Staining and Mycelial Morphology

The morphological changes and cell membrane integrity of *A. alternata* mycelia were assessed as per Li et al. [34] with minor modifications. In brief, three disks of *A. alternata* were inoculated into 0.15 L PDB medium and cultured on a constant temperature shaker (150× *g* at 28 °C) for 3 d. Then, the wet mycelia were collected and transferred to fresh PDB containing 50 mL L^−1^, 100 mL L^−1^, and 200 mL L^−1^ AF. The PDB-treated mycelia were used as a negative control. After incubation for 12 h, the mycelial morphology changes were observed under an optical microscopy (LEICA ICC50 W, Leica Microsystems Co., Ltd., Shanghai, China). To further investigate the cell membrane integrity of *A. alternata*, the mycelial suspension of *A. alternata* was centrifuged (10,000× *g*, 10 min, 4 °C) and washed three times with 0.1 M phosphate-buffered saline (PBS, pH 7.0). Subsequently, mycelia were stained with 5 mg L^−1^ PI fluorescent dye for 30 min in the dark. The mycelia were then washed twice with 0.1 M PBS and visualized and photographed under a laser scanning confocal microscope (NE 910-FL, Ningbo Yongxin Optics Co., Ltd.,Ningbo, Zhejiang, China).

### 2.9. SEM

To further detect morphological changes in *A. alternata* mycelia, untreated mycelia and mycelia treated with 200 mL L^−1^ AF for 12 h were washed three times with 0.1 M PBS (pH 7.0) and fixed using 2.5% (*v/v*) glutaraldehyde at 4 °C for 24 h. The fixed mycelia were dehydrated using a graded series of ethanol (30%, 50%, 70%, 80%, 90%, and 100%) for 15 min, respectively. Subsequently, the dehydrated mycelias were critical-point-dried with CO_2_ and then coated with gold. The samples were finally scanning via the scanning electron microscope (SU-8010, Hitachi, Tokyo, Japan) operating at 3.0 kV at 10,000×, 4000×, 2000×, and 1000× levels of magnification.

### 2.10. Electrical Conductivity

Three 6-mm mycelial disks of *A. alternata* were transferred to 0.25-L flasks containing 0.15 L PDB. The flasks were placed on a constant temperature shaker (150× *g* at 28 °C). After incubation for 7 d, the mycelia were washed three times with sterile water and filtered in a vacuum for 10 min. Then, 1 g fresh mycelia was suspended in 30 mL PDB medium containing 50 mL L^−1^, 100 mL L^−1^, and 200 mL L^−1^ AF. Sterile water without AF was used as a control. After incubation for 3, 6, 12, 24, 48, and 72 h, the electrical conductivity of the mycelial suspensions was measured with a conductivity meter (DDSJ-319L, Shanghai INESA Scientific Instruments Co., Ltd., Shanghai, China).

### 2.11. Intracellular Content Release

The intracellular contents released into the supernatant from *A. alternata* were detected using previous method [35] with minor modifications. Mycelia were cultured as described in Section 2.10. In brief, 1 g mycelia was suspended in 0.03 L PDB medium containing 50 mL L^−1^, 100 mL L^−1^, and 200 mL L^−1^ (*v*:*v*) AF. PDB medium without AF was used as a negative control. The mixtures were placed on a constant temperature shaker (150× *g* at 28 °C), and the supernatants cultured for 3, 6, 12, 24, 48, or 72 h were collected by centrifugation at 8000× *g* for 5 min. Subsequently, 200 μL supernatant was injected into the 96-well ultraviolet microplates (SuperMax 3100, Shanghai Flash Spectrum Biological Technology Co., Ltd., Shanghai, China). The release of the intracellular contents into the supernatants was expressed in terms of the optical density at 260 nm (for nucleic acids) and 280 nm (for protein).

### 2.12. Chitinase and β-1,3-Glucanase Activities

To assess the effect of AF on mycelial enzyme activities of chitinase and β-1,3-glucanase, the mycelia of *A. alternata* were inoculated into 150 mL PDB medium containing 50 mL L^−1^, 100 mL L^−1^, and 200 mL L^−1^ AF and cultured on a rotary shaker (150× *g* at 28 °C). After incubation for 3, 6, 12, 24, 48, or 72 h at 28 °C, the mycelia were collected by centrifugation at 10,000× *g* for 10 min at 4 °C. The chitinase and β-1,3-glucanase activities of *A. alternata* mycelia were determined spectrophotometrically using commercially available kits (Beijing Solarbio Science & Technology Co., Ltd., Beijing, China) as per the manufacturer’s instructions. PDB medium without AF was used as a negative control. All experiments were performed in triplicate. A unit of chitinase enzyme activity is defined as the production of 0.001 mol N-acetamidoglucose by decomposing chitin per kg of tissue per h. A unit of β-1,3-glucanase enzyme activity is defined as the production of 1 g reducing sugar per kg of tissue per h. The activity of chitinase enzyme and β-1,3-glucanase enzyme was expressed as U kg^−1^.

### 2.13. Aseptic Filtrate Component Identification by GC–MS

The components of the AF were identified by GC–MS (Agilent, 7890B/LECO, Pegasus BT, Santa Clara, California, USA) according to previous method [36]. Gas chromatography was performed via a DB-5MS capillary column (30 m × 250 μm i.d., 0.25-μm film thickness, Agilent J & W Scientific, Folsom, CA, USA), and the derivatives were separated at a constant flow rate of 1 mL/min helium. One microliter of the sample was injected in split mode at a 1:10 split ratio by the autosampler. The injection temperature was 280 °C. The temperatures of the transfer line and ion sources were 320 °C and 230 °C, respectively. The temperature rising program taken 50 °C as the initial temperature for 0.5 min, rose to 320 °C at the rate of 15 °C/min, and then maintenance at 320 °C for 9 min and then maintenance at 320 °C for 9 min. Mass spectrometry was performed using a full-scan method at a scan rate of 10 spec/s, electron energy of −70 V, and a solvent delay of 3 min.

### 2.14. In Vivo Antifungal Activity Assays

A uniform wound (2-mm deep × 7-mm diameter) was created at the fruit equator using a sterilized punch. Then, 200 μL of different concentrations of AF (50 mL L^−1^, 100 mL L^−1^, and 200 mL L^−1^) was pipetted into each wound. An equal volume of sterile water was used as a control group. After airdrying, a disk (2-mm deep × 7-mm diameter) of *A. alternata* was inoculated onto each wound. Subsequently, the fruit were placed in a sterile crisper and kept in an artificial climate cabinet (HWS-436, Ningbo Jiangnan Instrument Factory Ltd., Ningbo, China) with a fixed 12 h of light and 12 h of photoperiod at 28 °C and 80% relative humidity. After incubation for 6, 9, and 12 d, the decay incidence and lesion diameter were measured using Image J software (National Institute of Health, Bethesda, MD, USA). The disease incidence (%) was calculated using the formula: (A1 − A2)/A1 × 100, where A1 and A2 represent the decayed areas in the treatment group and control group, respectively. The experiment was performed in triplicate.

### 2.15. Statistical Analyses

All data were statistically analyzed using Excel 2010 and SPSS version 25 (SPSS Inc., Chicago, IL, USA). The one-way ANOVA was performed as per Duncan’s multiple range test to determine the significant difference at *p* < 0.05. Charts were plotted with Origin 2021.

## 3. Results

### 3.1. Screening and Identification of Antagonistic Fungi

**Figure 1:**
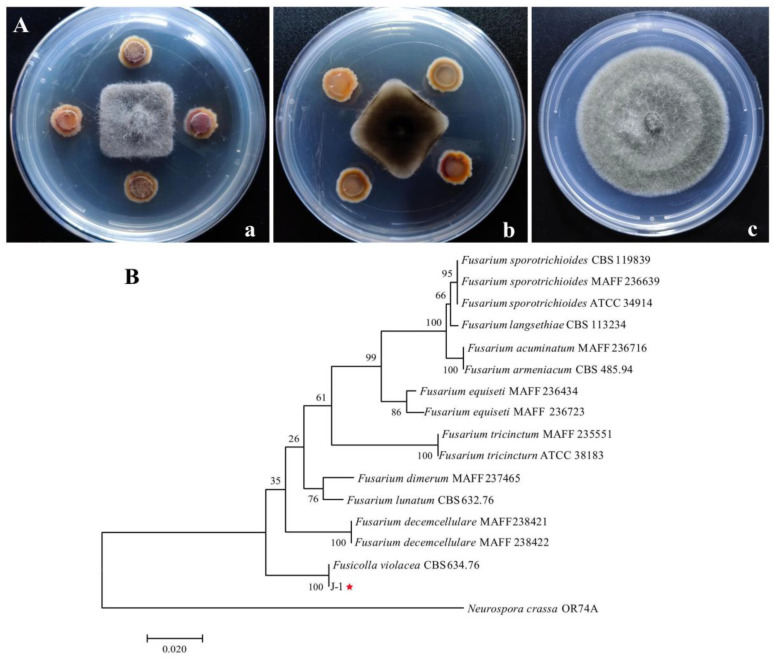
Identification of strain J-1 with high antagonistic activity against *A. alternata*. (**A**) In vitro inhibition activity; (**a**) Surface view of colony; (**b**) Back view of colony; and (**c**) Control. (**B**) A phylogenetic tree based on 18S rDNA and β-tub sequences constructed using the maximum likelihood (ML) method in MEGA 7.0 software with bootstrap values based on 1000 replications. Note: the red star indicate antagonistic strain.

**Figure 2:**
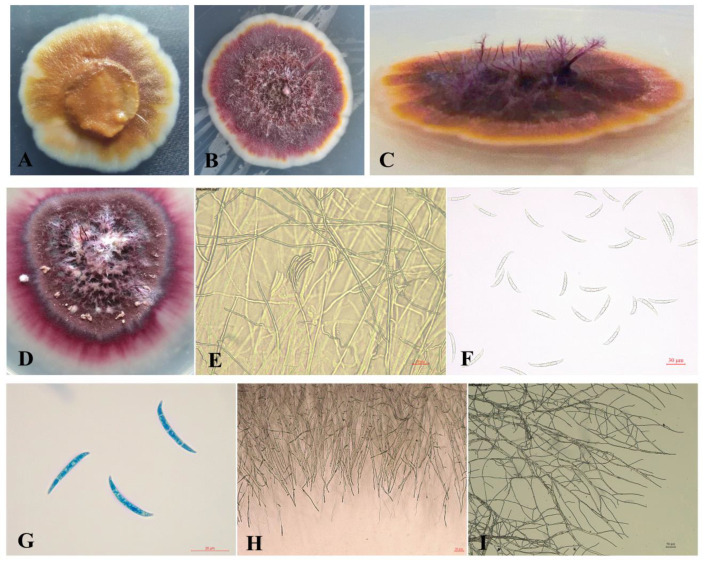
The morphology of antagonistic fungus J-1 grown on PDA medium for 10 d. (**A**) Slimy or yeast-like colony. (**B**,**C**) Crown mycelia. (**D**) Sticky spores group on PDA medium. (**E**) Phialidic conidi-ogenous cells. (**F**,**G**) Conidiophores. (**H**,**I**) Mycelial morphology. The scale bar in Figure 2E, Figure 2F, Figure 2G, Figure 2H and Figure 2I are 20 μm, 30 μm, 20 μm, 50 μm, and 50 μm respectively.

Eight strains of fungi were obtained from *Actinidia cinensis* “Hongyang.” Among them, only one fungus, labeled J-1, showed strong inhibitory activity against *A. alternata*, and the inhibition effect on mycelia growth was about 66.1% in the dual culture experiment (Figure 1A). The strain J-1 was then selected for further analysis. The colonies of the strain J-1 grew very slowly (2.013 mm d^−1^ at 28 °C) and were usually slimy or yeast-like in appearance with few or no aerial mycelia. The new colonies cultured on PDA were white or yellow, whereas the old colonies (cultured for 15 d) were burgundy with a white fringe. In some cases, the middle mycelia of the colonies were crown-shaped. Conidia were produced on water agar containing carnation leaf pieces, and the conidiogenous cells were phialidic. Under optical microscopy, the conidia appeared slightly curved, with blunt and occasionally hooked apical cells and poorly developed basal cells. There were few small conidia, and the large, mature conidia were usually three-septate and measured 29.00~46.86 μm × 3.80~5.90 μm (Figure 2).

To identify the strain J-1, the sequences of the 18S rDNA (610) and *β-tub* (819) were amplified by PCR and then submitted to GenBank database with the accession numbers of 18S rDNA (OK318004) and *β-tub* (OK318005), respectively. The results showed that the 18S rDNA sequence of strain J-1 exhibited a 100% identity with *Fusicolla violacea* (AB586903). The *β-tub* sequence of strain J-1 showed 100% identity with that of *Fusicolla violacea* (AB587045). A multigene phylogenetic tree was constructed using the maximum likelihood (ML) method in the MEGA 7.0 software. Phylogenetic analysis further confirmed that the strain J-1 and *Fusicolla violacea* were clustered together (Figure 1B). Detailed morphological studies and sequence analyses confirmed that the strain J-1 belonged to *Fusicolla violacea*.

### 3.2. Aseptic Filtrate Effects on the Mycelial Growth of A. alternata

**Figure 3:**
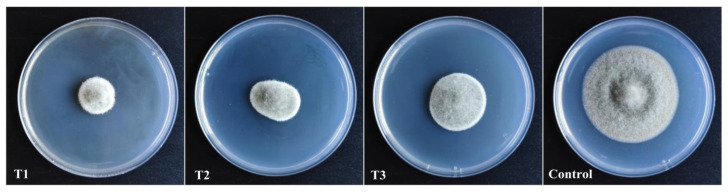
Antifungal activity of different concentration aseptic filtrates against *A. alternata* on PDA medium after 5 d. T1 (200 mL L^−1^); T2 (100 mL L^−1^); and T3 (50 mL L^−1^); Control (Sterile water treatment).

To investigate the inhibitory effect of the AF against *A. alternata* in vitro, the inhibition rate of the mycelial growth of *A. alternata* was assayed following AF treatment. The results showed that the inhibitory activity gradually increased with increasing AF concentration (Table 1 and Figure 3). At a 200 mL L^−1^ (*v*:*v*) AF concentration, the mycelial growth of *A. alternata* was significantly suppressed, with an inhibition rate of 66.8%.

### 3.3. Aseptic Filtrate Effects on the Conidia Germination of the Pathogen

**Figure 4:**
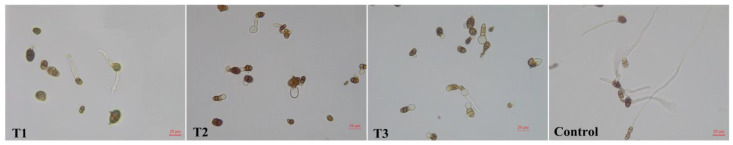
Determination of conidial germination of *A. alternata* treated with different concentration aseptic filtrates after 12 h. T1 (200 mL L^−1^); T2 (100 mL L^−1^); and T3 (50 mL L^−1^); Control (Sterile water treatment).

The conidia germination rate of *A. alternata* was assessed after AF treatment. The results indicated that the conidia germination of *A. alternata* was gradually inhibited along with increasing AF concentration (Table 2). Compared with 91.4% conidia germination in the control group, the conidia germination was 71.1%, 38.7%, and 18.4% at the AF concentrations of 50 mL L^−1^, 100 mL L^−1^, and 200 mL L^−1^, respectively. After treatment with AF, the conidia could not germinate normally, and the germ tubes that had germinated were enlarged and spherical or oval (Figure 4); as a result, the germ tubes could not extend normally, which indicated the decrease in infection ability. By contrast, the mycelia in the control group were uniformly thin and long with smooth tops.

### 3.4. Antifungal Spectrum of the Strain J-1 and Its Aseptic Filtrate 

**Figure 5:**
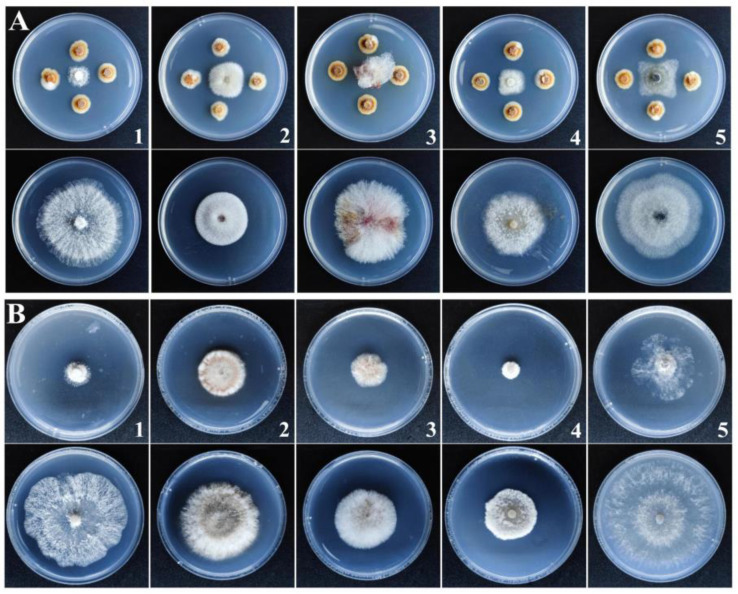
Antifungal spectrum of strain J-1 (**A**) and 200 mL L^−1^ aseptic filtrate (**B**) against five phytopathogenic fungi. (1. *Diaporthe eres*, 2. *Epicoccum sorghinum*, 3. *Fusarium graminearum*, 4. *Phomopsis* sp., and 5. *Botryosphaeria dothidea*).

To assess whether the strain J-1 and its AF had broad-spectrum antifungal activity, five fungal pathogens were selected for inhibition assay. In the dual culture experiment, the strain J-1 could significantly suppress the mycelial growth of all tested pathogens, with inhibition rates ranging from 34.4% to 75.1%. Among these, the mycelial growth of *Diaporthe eres* was significantly suppressed by the strain J-1, with an inhibition rate of 75.1% (Table 3 and Figure 5A). The mycelial growth of all tested pathogens was inhibited in PDA plates containing 200 mL L^−1^ (*v*:*v*) AF, and the inhibition rates ranged from 42.7% to 75.2% (Table 3 and Figure 5B).

### 3.5. Aseptic Filtrate Stability

**Figure 6:**
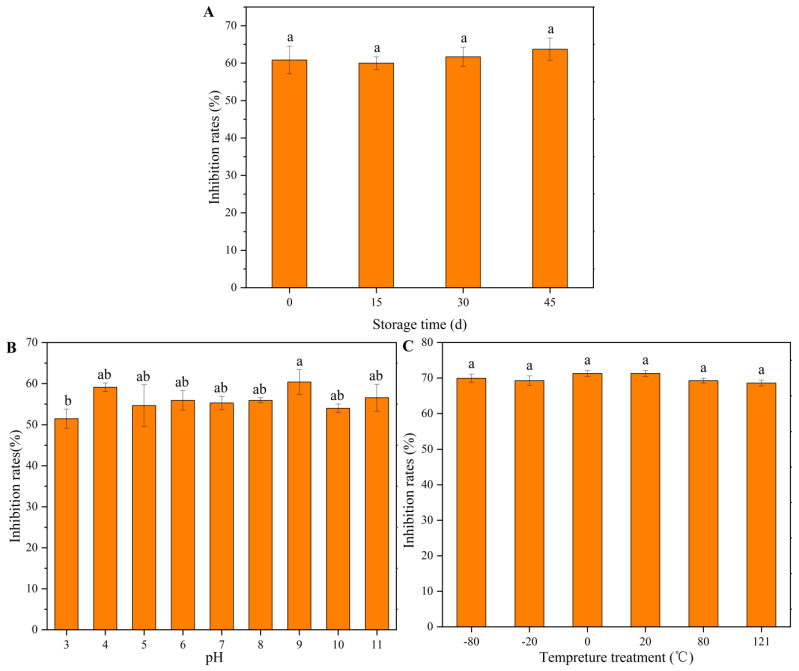
Stability of aseptic filtrate treated with different storage time (**A**), temperature (**B**), and pH values (**C**). Note: the aseptic filtrate concentration was 200 mL L^−1^. Numerical values were expressed as mean ± SE of triplicates. The same lowercase letters indicate no significant difference between treatments (*p* < 0.05).

The effects of different temperatures, pH, and storage times on the antifungal activities of the AF were evaluated. The results showed that different temperature treatments had no effect on the antifungal components produced by the strain J-1. Different pH values showed little effect on the antifungal activities of the AF. The AF still showed inhibitory activity against *A. alternata* after 45 d of storage at room temperature (Figure 6).

### 3.6. PI Staining and Mycelial Morphology

**Figure 7:**
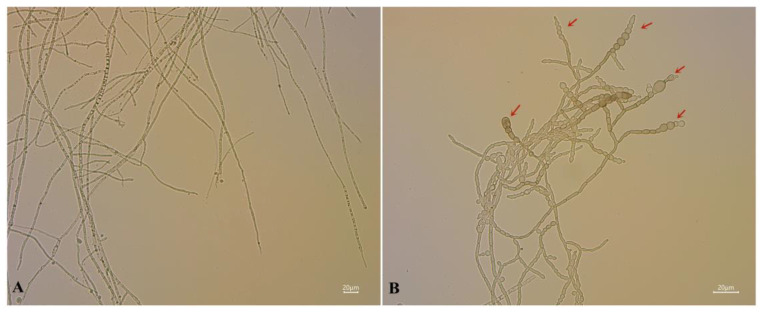
Aseptic filtrate effects on *A. alternata* mycelial morphology. (**A**) Untreated cells after 12 h. (**B**) Cells treated with 200 mL L^−1^ aseptic filtrate after 12 h. Note: The position indicated by the red arrows indicates the mycelial spherical protrusions.

**Figure 8:**
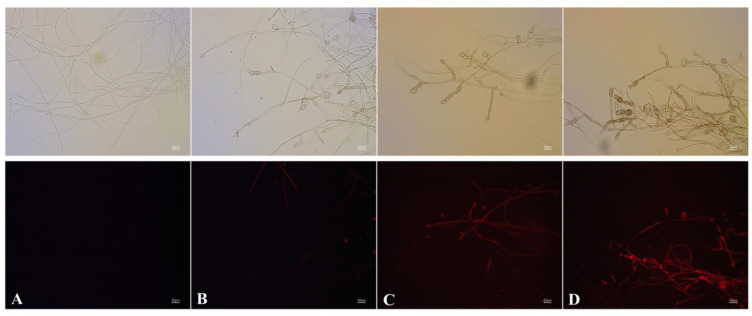
Effect of aseptic filtrate on *A. alternata* cell membrane integrity analyzed by PI staining. (**A**) Untreated cells after 12 h. (**B**–**D**): Cells treated with 50 mL L^−1^ (**B**), 100 mL L^−1^ (**C**), and 200 mL L^−1^ (**D**) aseptic filtrate after 12 h. The scale bars in Figure 8A–D are 20 μm.

Changes in the mycelial morphology of *A. alternata* after AF treatment were observed under optical microscopy. After treatment with 200 mL L^−1^ AF for 12 h, the number of *A. alternata* mycelial branches were increased compared with the control group. A large number of clusters of spherical protrusions occurred in the middle or on the tips of the mycelia (Figure 7). In addition, the number of spherical protrusions increased with the increasing concentration of AF, leading to the abnormal growth of mycelia (Figure 8). PI, a fluorescent dye, was chosen to determine the cell membrane integrity of *A. alternata* after exposure to AF. PI enters damaged mycelia and combines with nucleic acids to produce fluorescence. Mycelia treated with AF displayed a red fluorescence signal, and the signal was positively correlated with the increasing concentrations of AF (Figure 8). These results indicated that the cell membranes of *A. alternata* were damaged after treatment with the AF of J-1.

### 3.7. SEM

**Figure 9:**
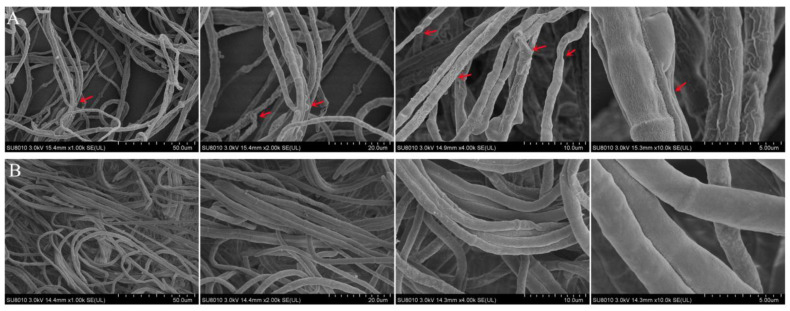
Scanning electron micrograph analysis of *A. alternata* mycelia ultrastructure morphology. (**A**) Mycelia treated with 200 mL L^−1^ aseptic filtrate after 12 h. (**B**) Untreated mycelia after 12 h. Note: The position indicated by the red arrows indicates the curved, deformed, or coarse hyphae.

To further confirm the antifungal action of the AF on *A. alternata*, SEM analysis was performed (Figure 9). Untreated mycelia were smooth and had a complete structure. By contrast, the mycelia of *A. alternata* treated with 200 mL L^−1^ AF for 12 h showed morphological damage. The mycelia were bent and malformed, and the surfaces were rough and protuberant. These results were in agreement with the changes in mycelial morphology observed under optical microscopy.

### 3.8. Cell Membrane Integrity of A. alternata with Aseptic Filtrate Treatment

**Figure 10:**
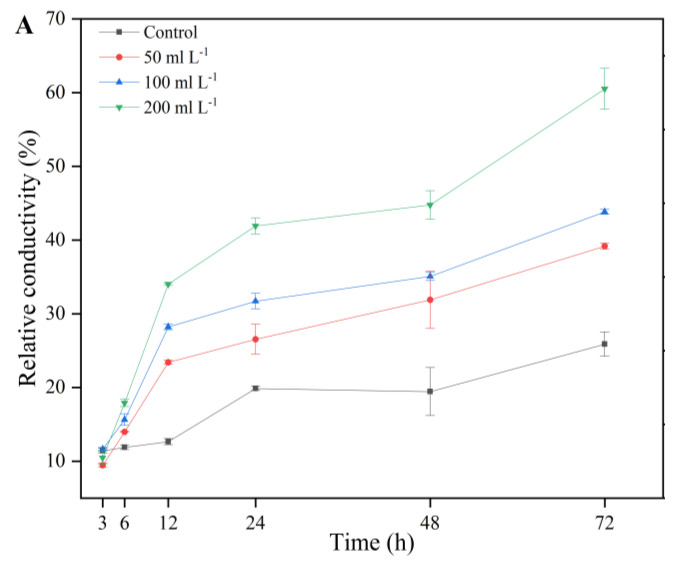

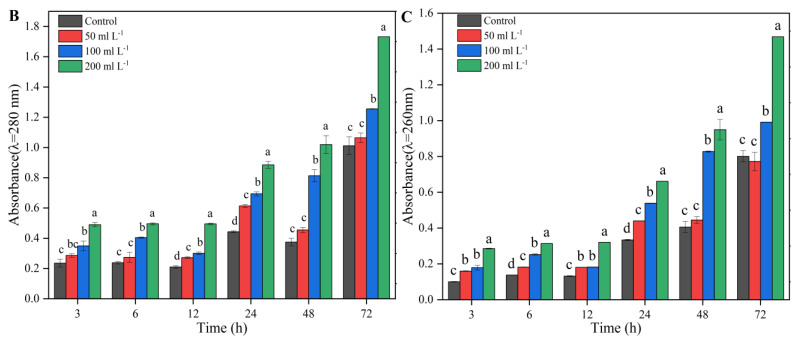
Detection of relative conductivity (**A**), nucleic acids leakage (**B**), and proteins leakage (**C**) in *A. alternata* cultures after treatment with 50 mL L^−1^, 100 mL L^−1^, and 200 mL L^−1^ aseptic filtrate for 3 h, 6 h, 12 h, 24 h, 48 h, and 72 h. Data are displayed as the mean ± SE. Bars with the same lowercase letters indicate no significant difference between treatments (*p* < 0.05).

To further study changes in the cell membrane of *A. alternata*, the relative conductivity and the leakage of nucleic acids (OD_260_) and proteins (OD_280_) of mycelial suspensions treated with AF were measured. The obtained results showed that the relative conductivity of the mycelial suspensions significantly increased with increasing AF concentration, indicating that the AF could damage the cell membranes of *A. alternata* (Figure 10A). The leakage of nucleic acids (Figure 10B) and proteins (Figure 10C) in *A. alternata* mycelia treated with AF was notably higher than that of the control group (*p* < 0.05), particularly after 24 h of treatment.

### 3.9. Chitinase and β-1,3-Glucanase Enzyme Activities

**Figure 11:**
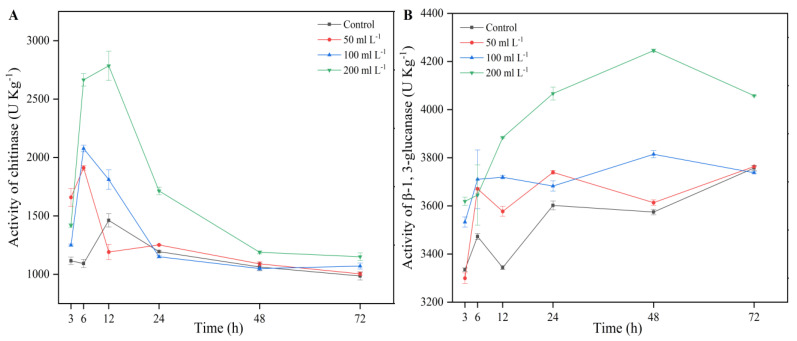
Detection of chitinase (**A**) and β-1, 3-glucanase (**B**) activities in cell wall of *A. alternata* after treatment with 50 mL L^−1^, 100 mL L^−1^, and 200 mL L^−1^ aseptic filtrate for 3 h, 6 h, 12 h, 24 h, 48 h, and 72 h. Data are displayed as the mean ± SE.

Along with changes in the mycelial morphology and membrane integrity of *A. alternata*, the chitinase and β-1,3-glucanase enzyme activities in the cell wall were investigated. Compared with the control, the chitinase and β-1,3-glucanase enzyme activities prominently increased (*p* < 0.05) in *A. alternata* cell walls when the concentration of AF increased. Chitinase activity increased significantly after treatment with 200 mL L^−1^ AF for 12 h and then decreased gradually (Figure 11A). The β-1,3-glucanase enzyme activity in *A. alternata* cell walls increased gradually and reached the maximum value at 48 h, suggesting that the cell wall was damaged (Figure 11B).

### 3.10. GC–MS Analysis

The potential bioactive components of the strain J-1 were identified using GC–MS. The metabolites were first identified according to the precise molecular weight (molecular weight error ≤ 30 ppm). As a result, a total of 103 different metabolites were identified against the Human Metabolome Database (http://www.hmhd.ca, accessed on 11 May 2021), METLIN (http://metlin.scripps.edu, accessed on 11 May 2021), Massbank (http://www.massbank.jp./, accessed on 18 May 2021), LipidMaps (http://www.lipidmaps.org, accessed on 20 May 2021), and mzCloud (https://www.mzcloud.org, accessed on 12 May 2021) according to the MS/MS fragmentation mode. Among these metabolites, 11 metabolic components showed antimicrobial activities according to previous studies. The detected components and their chemical structures are shown in Table 4.

### 3.11. Aseptic Filtrate Effects on Controlling the Decay of Kiwifruit

**Figure 12:**
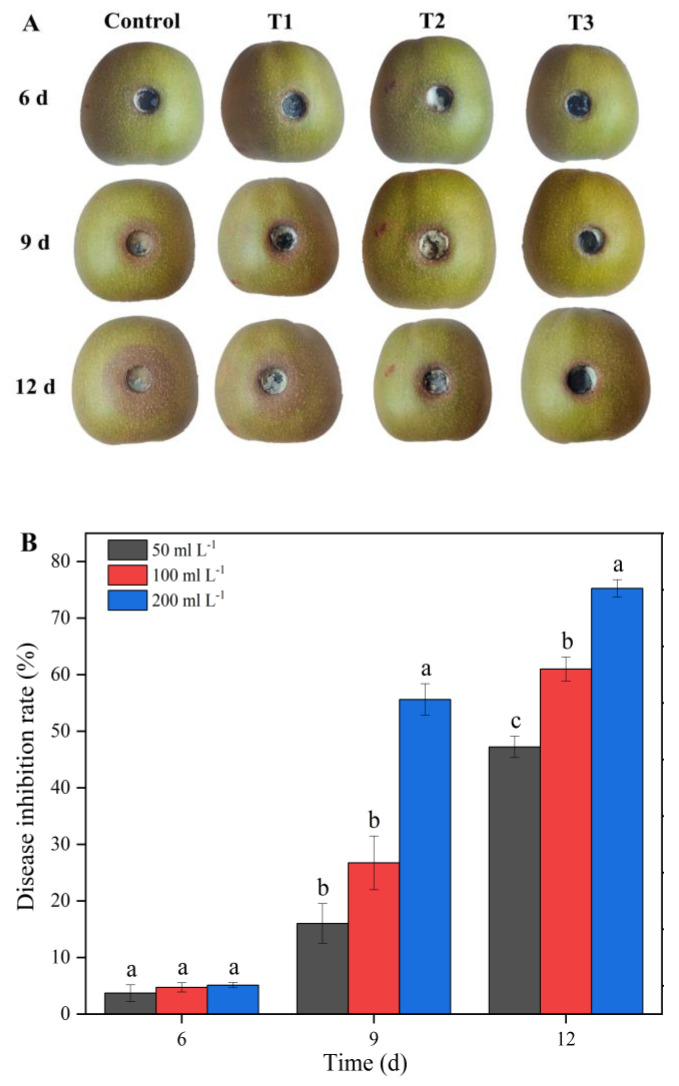
In vivo effect of aseptic filtrate on disease extension in kiwifruit caused by *A. alternata*. (**A**) Disease extension symptom in kiwifruit after treatment with 50 mL L^−1^ (T1), 100 mL L^−1^ (T2), and 200 mL L^−1^ (T3) aseptic filtrate for 6, 9, and 12 d. (**B**) Disease inhibition rate after treatment with different concentration aseptic filtrates. Data are displayed as the mean ± SE. Bars with the same lowercase letters indicate no significant difference between treatments (*p* < 0.05).

To confirm the biological control effect of the AF on *A. alternata* in vivo, the AF of the strain J-1 was used to alleviate the severity of kiwifruit decay caused by *A. alternata* (Figure 12). After 5 d of storage, no significant decay was detected in the kiwifruit treated with different concentrations of AF. After 9 d of storage, lesions began to appear in the fruit treated with different concentrations of AF other than 200 mL L^−1^ treatment. As the storage time increased to 12 d, the diameter of the lesions in the control group was increased compared with that in the treatment groups. The disease inhibition rates were 47.2% for 50 mL L^−1^ AF, 64.6% for 100 mL L^−1^ AF, and 75.3% for 200 mL L^−1^ AF. Therefore, 200 mL L^−1^ AF exhibited excellent biocontrol effects during storage.

## 4. Discussion

Kiwifruit is susceptible to infection by various pathogens during storage and transport, resulting in serious deterioration in fruit quality and considerable economic losses [37]. *A. alternata*, one of the most pathogenic fungi, can cause postharvest rot and black rot in harvested kiwifruit [38,39], although it is not as epidemic as *Phomopsis* sp. And *Botryosphaeria dothidea* [2]. At present, the control of kiwifruit rot primarily relies on synthetic fungicides; however, the long-term use of synthetic fungicides carries concerns regarding food safety and environmental pollution. Therefore, it is important to find alternative methods to control postharvest diseases. In recent years, biological control has received widespread attention for its ability to promote crop growth, increase beneficial microbial populations, reduce the use of chemical pesticides, and effectively control crop diseases [40]. In general, competition for nutrients and space are the main inhibitory mechanisms of microbial antagonists. Non-pathogenic *Fusarium oxysporum* and *Pseudomonas fluorescens* could inhibit Fusarium wilt disease by competing with pathogenic *Fusarium oxysporum* for nutrients such as carbon and iron [41]. The production of antibiotics, direct parasitism, and induced resistance are other inhibitory mechanisms by which biological control agents suppress the spread of pathogens [42]. Tetramycin, an environmentally friendly antibiotic, produced by *Streptomyces hygrospinosus* var. *Beijingensisis*, has been widely used in the prevention and control of plant diseases due to its high efficiency and low toxicity [43]. Arbuscular mycorrhizal fungi (AMF) are symbiotic fungi living with plant roots which can promote crop growth and improve its resistance to pathogens [44]. Previous studies reported that the plant growth promoting rhizobacteria (PGPR) could improve plant resistance against pathogens and promote crop growth [45]. Kalantarl et al. confirmed that the mixtures of *Bacillus*, *Pseudomonas*, and *Rhizobium* exhibited a synergistic impact by producing various beneficial substances, which could effectively suppress the Fusarium root rot and improve bean yield [46].

In the present study, we isolated an endophytic fungus, *Fusicolla violacea* J-1, with strong antifungal activity against *A. alternate*, from *Actinidia cinensis* “Hongyang.” We evaluated, for the first time, the antifungal effect and potential mechanism of this strain against *A. alternata.* The strain *Fusicolla violacea* J-1 can be isolated from kiwifruit, indicating that it has good potential to parasitize kiwifruit. Moreover, *Fusicolla violacea* (*Fusarium merismoides* var. *Violaceum*) is one of the varieties of *Fusarium merismoides* Corda [47]. Studies have proven that *Fusarium merismoides* Corda could parasitize on the oospores of *Pythium ultimum*, which may be used as a biocontrol agent [48]. The endophytic capabilities of fungi are sometimes correlated with related fungal species or genera. Based on this, we think that *Fusicolla violacea* also has well endophytic capabilities on other pathogens or plants. Endophytic fungi can inhabit in the tissues of host without causing diseases [49]. Therefore, *Fusicolla violacea* has the potential to be developed as a biocontrol agent. Thus far, *Fusicolla violacea* has not been reported as an antagonistic fungus to control plant diseases. Previous studies have reported other microbial control agents for kiwifruit rot, including the antagonistic yeasts *Candida oleophila, Debaryomyces hansenii*, and *Hanseniaspora uvarum* [50,51,52]. Kurniawan et al. [53] reported that antagonistic bacteria from the *Bacillus* and *Pseudomonas* genera reduced the mycelial growth of *Botrytis cinerea* and *A. alternata*, thereby reducing the rot incidence and severity of gray mold and *Alternaria* rot on highbush blueberry (*Vaccinium corymbosum* L.) fruit. Moreover, rhizobacterial strains such as *Burkholderia cenocepacia* VBC7 and *Pseudomonas poae* VBK1 were reported to exhibit strong antagonistic effects against aloe vera leaf spot disease caused by *A. alternata* [54].

The results we obtained showed that *Fusicolla violacea* inhibited the growth of *A. alternata* by > 60% in the dual culture experiment (Figure 1A). The edge of the pathogenic fungi’s mycelia became black, indicating that the bioactive components produced by the strain J-1 had destroyed the mycelia and not just inhibited mycelium growth via space and nutrient competition. The AF could efficiently inhibit the extension of *A. alternata* in vivo, with the increasing inhibition observed with increasing AF concentrations (Figure 12). Consistent with the results of antagonistic activity in vitro, the AF significantly suppressed the mycelial growth of *A. alternata*. The 200 mL L^−1^ AF concentration showed the highest antagonistic activity, with an inhibition rate of 66.8%. Moreover, a large number of clusters of spherical protrusions appeared at the mycelium tips (Figure 7), which was in agreement with the changes in mycelial morphology observed via SEM (Figure 9). The conidia germination test showed that the germ tubes could not extend normally and developed into round or oval bubbles (Figure 4), which indicated the decrease in infection ability. Similar observations were reported in a previous study [55]. To further clarify the inhibitory mechanism of the AF against *A. alternata*, the integrity of the cell membranes and activities of key enzymes related to the cell wall were measured. Compared with the control, the relative conductivity and the leakage of nucleic acids and proteins of the mycelial suspensions significantly increased with increasing AF concentration (Figure 10), indicating that the integrity of the cell membrane of the pathogen had been damaged. This was further confirmed by the PI staining results (Figure 8). In addition, the cell wall-related enzyme activities, chitinase and β-1,3-glucanase, increased notably after treatment with the AF (Figure 11), suggesting that the cell walls of the pathogen were damaged.

To evaluate the antifungal spectrum of the strain J-1, five pathogenic fungi were selected. The strain J-1 and its aseptic filtrate could significantly suppress the mycelial growth of five pathogens in the dual culture tests. These results may be related to the antagonistic fungus producing a variety of bioactive substances. In the present study, 11 antimicrobial metabolic substances produced by the strain J-1 were identified using GC–MS. It has been reported previously that nonanoic acid produced by *Trichoderma harzianum* inhibited the spore germination and mycelial growth of two pathogens of cacao [56]. Jang et al. [57] also reported nonanoic acid’s antifungal potential. Sari et al. [58] showed that 4-hydroxyphenylacetic acid has antioxidant potential, whereas resorcinol and its derivatives were demonstrated to have antifungal activities by Karunanayake et al. [59]. Antifungal resorcinols have been implicated in the quiescence of *C. gloesoporioides* and *A. alternata* on mango [60]. Oxalic acid, identified from the volatile organic compounds of *Metarhizium anisopliae* Ma70, has been reported as the most promising antifungal agent against *B. cinerea*, which causes apple gray mold [61]. Hexadecanoic acid and octadecane have been demonstrated to show antifungal activity against *Colletotrichum gloeosporioides* [62]. Deepthi et al. [63] confirmed that 10-octadecenoic acid and heptadecanoic acid produced by *Lactobacillus plantarum* MYS6 are major antifungal compounds against a fumonisin-producing fungus, *Fusarium proliferatum* MYS9. Liu et al. [64] reported a novel antiyeast lactic acid bacteria strain, *Lactobacillus parafarraginis* ZH1, that produces hexadecanoic acid. Heneicosane was found to exhibit excellent antimicrobial activity against *Streptococcus pneumoniae* and *Aspergillus fumigatus* [65]. Barupal et al. [66] identified six constituents from the leaf extract of *Lawsonia inermis* and confirmed that docosane and octacosane exhibited antifungal activity against *Curvularia lunata*. Hydroxyurea is a well-established inhibitor of ribonucleotide reductase and causes cell death and DNA damage in various organisms [67]. Based on the above studies, we speculated that the broad-spectrum antagonistic activity of the strain J-1 was related to the production of various metabolites (Table 4), including alkanes, fatty acids, phenols, and organic acids. To the best of our knowledge, this is the first time that nonanoic acid, 4-hydroxyphenylacetic acid, resorcinol, and hydroxyurea have been examined from *Fusarium* sp. that may play a key role in suppressing pathogens. However, it is not clear whether one or more metabolites have negative effects on mycelial growth, conidia germination and mycelial morphological changes of *A. alternarta*. Therefore, further work is necessary to identify the key bioactive metabolites produced by *Fusicolla violacea* and explore their effect for pathogen inhibition and antifungal mechanism. This study enriches our knowledge of secondary metabolites derived from *Fusarium* sp., and these bioactive substances exhibit the potential value of being developed into biological agents.

## 5. Conclusions

In this study, a new antagonistic strain, *Fusicolla violacea* J-1, isolated from *Actinidia cinensis* “Hongyang,” exhibited strong antifungal activity against *A. alternata* both in vitro and in vivo. The AF of the strain J-1 significantly suppressed the mycelial growth and conidia germination of *A. alternata* and caused mycelial morphological changes. The AF of *Fusicolla violacea* J-1 had a broad antifungal spectrum against five pathogens. The results showed that temperature, pH, and storage time had little effect on the antifungal activity of the AF. The AF significantly improved crucial cell wall enzyme activity; destroyed the integrity of the cell membrane; caused the leakage of nucleic acids, protein, and other substances; and caused cell death. Eleven bioactive substances were identified in the AF of *Fusicolla violacea* J-1. The results of this study indicate that strain J-1 could be used as a potential biological resource for controlling postharvest fruit decay.

## Figures and Tables

**Table 1:** Inhibitory activity of aseptic filtrate against *Alternaria alternata*.

Treatment	Colony Diameter (mm)	Inhibition Rate (%)
200 mL L^−1^ AF	18.50 ± 0.21 d	66.8 ± 0.4 a
100 mL L^−1^ AF	25.67 ± 0.26 c	53.9 ± 1.7 b
50 mL L^−1^ AF	33.00 ± 0.95 b	40.7 ± 0.5 c
Control	55.67 ± 0.22 a	-

Numerical values were expressed as mean ± standard error (SE) of triplicates. Different lowercase letters represented a significant difference (*p* < 0.05).

**Table 2:** Effect of aseptic filtrate on conidia germination of *Alternaria alternata*.

Treatment	Conidia Germination Rate (%)	Inhibition Rate (%)
200 mL L^−1^ AF	18.4 ± 4.2 d	80.0 ± 5.6 a
100 mL L^−1^ AF	38.7 ± 5.7 c	57.8 ± 4.3 b
50 mL L^−1^ AF	71.1 ± 8.1 b	22.5 ± 7.8 c
Control	91.4 ± 1.4 a	-

Numerical values were expressed as mean ± standard error (SE) of triplicates. Different lowercase letters represented a significant difference (*p* < 0.05).

**Table 3:** Antifungal spectrum of strain J-1 and its aseptic filtrate.

Number	Pathogens	Disease	Inhibition Rate by Strain J-1 (%)	Inhibition Rate by AF (%)
1	*Diaporthe eres*	Black spot on kiwifruit	75.1 ± 0.9 a	75.23 ± 0.80 a
2	*Epicoccum sorghinum*	Leaf Sheath and Spot on Maize	34.4 ± 1.7 d	46.0 ± 0.8 c
3	*Fusarium graminearum*	Fusarium head blight on wheat	62.4 ± 0.7 b	42.7 ± 2.8 c
4	*Phomopsis* sp.	Soft rot on kiwifruit	56.6 ± 1.4 b	69.2 ± 0.9 b
5	*Botryosphaeria dothidea*	Soft rot on kiwifruit	52.0 ± 5.2 c	46.2 ± 2.1 c

Numerical values were expressed as mean ± standard error (SE) of triplicates. Different lowercase letters represented a significant difference (*p* < 0.05).

**Table 4:** Bioactive molecules identified from the aseptic filtrate of strain J-1 by GC–MS analysis.

Number	Compound	Structure	RT	Molecular Formula
1	Nonanoic acid	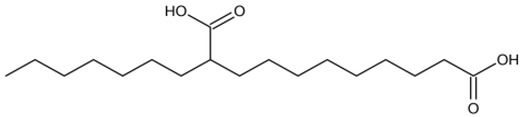	7.837	C_9_H_18_O_2_
2	4-Hydroxyphen-ylacetic acid	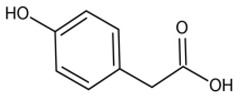	9.606	C_8_H_8_O_3_
3	Resorcinol	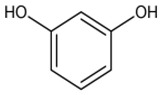	8.119	C_6_H_6_O_2_
4	Oxalic acid	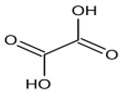	6.002	C_2_H_2_O_4_
5	Hexadecanoic acid	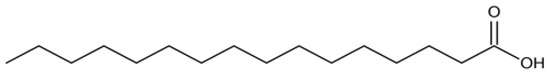	11.754	C_16_H_32_O_2_
6	Heptadecanoic acid	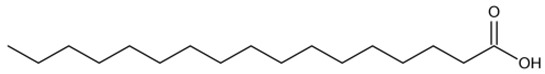	12.213	C_17_H_34_O_2_
7	10-Octadecenoic acid	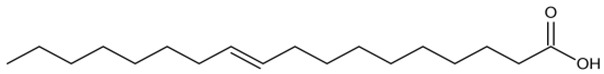	12.553	C_18_H_34_O_2_
8	Octadecane	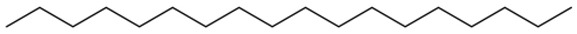	11.989	C_18_H_38_
9	Hydroxyurea	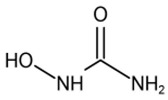	6.928	CH_4_N_2_O_2_
10	Heneicosane	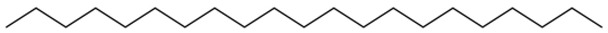	12.931	C_21_H_44_
11	Docosane	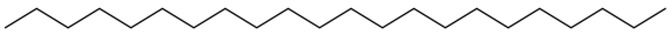	13.256	C_22_H_46_

## Data Availability

The datasets generated and/or analyzed during the study are available from the corresponding author upon reasonable request.
